# Genitourinary and Syphilitic Diseases

**Published:** 1903-01

**Authors:** Byron H. Daggett

**Affiliations:** Buffalo, N. Y.


### Genitourinary and Syphilitic Diseases.

CONDUCTED BY BYRON H. DAGGETT, M. D., Buffalo, N. Y.

### RETENTION OF URINE AND ITS RELIEF.

BRINTON, JOHN H., (*Therapeutic Gazette*, November 15, 1902,) says retention of urine is of frequent occurrence and its relief is some times readily and easily effected, at other times it is most difficult, demanding the highest skill.

Prior to the classic utterances of Sir Henry Thompson, text-book instructions were vague and the choice of instruments was at haphazard. Little attention was paid to the differing pathological causes of retention and methods were governed by the "rule of thumb." In later years there has been more careful study and investigation of the varieties and nature of pathological changes leading to retention as well as a better selection of suitable instruments, and full appreciation of their use.

*Causes of Retention.*—Mechanical, such as ligature or ring around the penis, a calculus or some solid substance in the canal, abnormal erotic freaks. Such obstructions must be removed. Retention may be dependent upon acute inflammatory engorgement of the prostate, caused by gonorrhea. Congestion of the lateral prostatic lobes may cause complete retention by pressure on the urethral canal. Some variety of soft catheter should be used, either the elbowed catheter of Mercier, made of silk or linen or rubber, or Nélaton's straight soft rubber instrument. [Tieman makes a soft rubber elbowed catheter—a very useful instrument.—ABSTRACTOR.]

The proximal end of the catheter should be soft so as not to bruise the swollen and tender urethral wall. The distal three-fourths of the catheter may be stiffened by inserting a small round whalebone to within 1½ to 2 inches above the eye, and fastened to the catheter by a thread about the outer end. The urethra should be injected with olive oil and the patient placed in a recumbent position. The catheter of Mercier should be pushed down with the beak projecting upward, and when soft resistance is felt some pressure is used to traverse the obstruction, the shank or body of the instrument made rigid as stated above. A new woven catheter possesses the necessary rigidity. These whalebones may be found in a ladies' trimming store; they are about the size of a large knitting needle and two feet long. The lower end of the whalebone should not come within two inches of the catheter eye and should be firmly fastened at the distal end of the catheter. Recurrence of the acute prostatic inflammation does not often happen.

*Retention produced by organic stricture.*—This usually follows a gonorrhea of long standing and develops gradually. After a time the catheter is occasionally used. Pain is caused by urination. Dribbling and straining occur with each effort to urinate; this continues until the urine cannot pass at all; then surgical aid is sought. Retention due to stricture demands immediate relief, requiring skill and tact on the part of the surgeon.

A great deal has been and even now is said concerning "impermeable or impassable stricture." Limiting the word "stricture" to the pathological obstruction of the urethra, the result of idiopathic cause, or gonorrhea, and excluding traumatic stricture following wounds or surgical operations, I do not believe that there is such a thing as impermeable or impassable stricture. As to the socalled impermeable variety, I believe the urine will always pass,—it may be in a tiny stream or drop by drop, but still it passes; in other words, the stricture is permeable. In regard to the much talked of "impassable" form of stricture, I cannot admit its existence, except in the traumatic variety of stricture. With this exception, wherever and whenever the urine passes downward along the urethra, an instrument of some sort can be passed upward into the bladder, provided, however, that the exigencies of the case do not limit the time for surgical manipulation. In this form of stricture there is always a channel,—it may be narrowed and contracted, but still it exists in continuity. On the other hand, in a traumatic stricture (if truly impassable) the urethra may have been lacerated or severed, and the ends of the canal superimposed or distorted and displaced. The continuity of the canal may have been interrupted to such a degree as to forbid the passage of instruments.

In dealing, then, with an organic idiopathic stricture, the result of an old gonorrhea, it is well for the surgeon to start with the idea that it is passable by an instrument,

I prefer filiform bougies for treating these strictures, over which the tunneled catheter of Gouley may be passed. The filiforms found in the stores are not as satisfactory as those I make from round whalebones of commerce. The ready-made filiforms may be made more flexible at the point by scraping with a knife. The olive-shaped tip of the home-made instrument can be shaped by scraping with a knife and polished with emery paper, tacked on some firm surface. Filiforms should have flexible, capillary and pliable extremities in order to detect and enter the minute openings of the strictured canal.

In treating stricture we assume that every organic stricture is due to the submucous deposit of organised lymph about some portion of the urethral circumference. This organised lymph constitutes the stricture, while the mucous membrane is puckered and drawn in by it and in such a stricture there is always a channel of some sort for the passage of urine in a greater or less quantity. The distal orifice of this narrow channel may be situated at any portion of the urethral circumference. It always exists and can always be penetrated by a filiform. If the probe point of the filiform can be made to enter the orifice of the channel, it will pass through and another can be passed beside it, thus enlarging the canal and preparing it for further surgical treatment. If more than one stricture is found, the filiform readily passes through into the bladder.

In addition to the filiforms the operator should have three or four Gouley tunneled sounds or catheters, in size from 8 to 16, French.

Before introducing the filiform inject olive oil into the urethra, draw the penis tense and pass the instrument with a rotary motion. If it will not readily pass the stricture, insert another and continue until half a dozen are introduced, and then manipulate one after another until the orifice is found.

If the opening is not found change position to the other side of the patient and withdraw the bundle of filiforms half an inch and begin again, pushing forward gently and consecutively each filiform in turn with a slight rotary motion. There is every chance, 99 in 100, that one will pass, then over this pass the tunneled sound or one or more of the filiforms. If the stricture be tight or irritable, it is well to tie in filiforms and leave them over night.

*Retention from Senile Enlargement of the Prostate.*—Senile enlargement of the prostate is due to abnormal development of its glandular and cellular structures and generally all portions of the prostate are simultaneously affected, although it may involve only one lobe, which will distort and render the course of the urethra tortuous. Frequently the enlargement of the prostate upward and backward causes a ridge or bar at the neck of the bladder forming a pouch, or receptacle for retaining urine, which stagnates and decomposes, often giving rise to the formation of calculi.

Symptoms are impeded, slow, frequent and delayed urination, loss of force, drip, urinous odor and staining of clothes, increased amount and frequent, nocturnal micturitions and residual urine.

If retention occurs it must be relieved by catheterisation. If the obstruction be due to swollen lateral lobes the Nélaton catheter, with a sufficient shank and a soft, pliant point should be used. For the projection of the third lobe an upturned tip or a soft beak catheter is suitable. The Mercier elbowed catheter of silk, linen or rubber possesses these qualities and if stiffened in the shank is suited to all cases of senile enlargement.

"Much of the difficulty of catheterisation can be overcome by putting your patient in a proper position, and by occupying a proper position yourself."—Pancost.

In a bad case tie in the catheter.

---

### NEPHROPEXY BY A NEW METHOD.

BEYER (*Pennsylvania Med. Jour.*, May, 1902,) after the usual incision and peeling of the fatty capsule, delivers the kidney through the wound and small rubber drainage tubes are passed through the perirenal fascia, one above and one below the renal vessels, drawn up around the kidney through the external wound and tied over a roll of gauze. These tubes remain in for three weeks and around them are formed connective tissue bands, which hold the kidney in place.

